# Procedural Software Toolkit in the Armamentarium of Interventional Therapies: A Review of Additive Usefulness and Current Evidence

**DOI:** 10.3390/diagnostics13040765

**Published:** 2023-02-17

**Authors:** Abdulaziz M. Al-Sharydah, Faisal Khalid BinShaiq, Rayan Ibrahim Aloraifi, Abdulrahman Abdulaziz Almefleh, Saud Abdulaziz Alessa, Adi Saud Alobud, Abdulmonem Mohammed AlSharidah, Abdulmajeed Bin Dahmash, Mohammad S. Al-Aftan, Bander Fuhaid Al-Dhaferi

**Affiliations:** 1 Diagnostic and Interventional Radiology Department, King Fahd Hospital of the University, Imam Abdulrahman Bin Faisal University, AlKhobar City 36277, Eastern Province, Saudi Arabia; 2 College of Medicine, King Saud bin Abdulaziz University for Health Sciences, Riyadh 14611, Riyadh Province, Saudi Arabia; 3 College of Medicine, King Fahd Hospital of the University, Imam Abdulrahman Bin Faisal University, AlKhobar City 36277, Eastern Province, Saudi Arabia; 4 Ad Diriyah Hospital, Ministry of Health, Riyadh 13717, Riyadh Province, Saudi Arabia

**Keywords:** embolization, imaging guidance, interventional radiology, oncology, targeting materials, thermal ablation

## Abstract

Interventional radiology is a fast-paced specialty that uses many advanced and emerging technological solutions. Several procedural hardware and software products are available commercially. Image-guided procedural software helps save time and effort in interventionist practice and adds precision to the intraoperative decisions made by the end user. Interventional radiologists, including interventional oncologists, have access to a wide range of commercially available procedural software that can be integrated into their workflow. However, the resources and real-world evidence related to such software are limited. Thus, we performed a detailed review of the current resources available, such as software-related publications, vendors’ multimedia materials (e.g., user guides), and each software’s functions and features, to compile a resource for interventional therapies. We also reviewed previous studies that have verified the use of such software in angiographic suites. Procedural software products will continue to increase in number and usage; these will likely be advanced further with deep learning, artificial intelligence, and new add-ins. Therefore, classifying procedural product software can improve our understanding of these entities. This review significantly contributes to the existing literature because it highlights the lack of studies on procedural product software.

## 1. Introduction

The increasing importance of minimally invasive interventional therapies, especially in oncology, dictates the use of well-structured and implementable radiological software technologies; thus, technological developments in interventional procedures have increased. Minimally invasive therapies require technological innovations to achieve better visualization, accurate localization of lesions, improved clinical outcomes, and optimized patient care [1,2,3].

In interventional radiology (IR), software enhancements are required to improve precision (i.e., reduce subjectivity bias among interventionalists), process data from multimodality imaging, and achieve fast real-time imaging [3,4].

Challenging therapeutic procedures, including tumor ablation and transarterial oncologic therapies, are associated with a considerable risk of complications, as well as increased procedural times and radiation doses. Navigational software tools, such as electromagnetic guidance systems and image fusion platforms, can help reduce the risk of complications from these procedures by enhancing the lesion-targeting process and minimizing the procedural times, risk of complications, and radiation doses. Consequently, procedural safety, efficacy, and patient outcomes can be improved [5,6].

Although several interventional systems and devices with different prototype software applications have been developed, they need to be investigated and optimized to be adequately applied in IR therapies. Interventionists typically rely on one imaging modality at a time and may even formulate a simulation or treatment plan remotely. Interventional radiological therapies are meant to be accurate, seamless, and minimally invasive; thus, appropriate integration of novel interventional software technologies into real-world interventional procedures may revolutionize IR [7].

Due to growing interest in the use of software in interventional procedures, the present review aims to provide a comprehensive and systematic overview of the utility of software programs in IR. Accordingly, 36 software programs are investigated, classified, and explored for available evidence. Furthermore, the current status of interventional software programs and potential future challenges are also discussed Our review contributes significantly to the existing literature because it highlights the lack of resources for thoroughly examining procedural product software.

## 2. Materials and Methods

A predefined protocol for this review was finalized on 2 December 2021. We comprehensively searched the MEDLINE, EMBASE, and PubMed databases for relevant articles published from database inception to 18 July 2022. Furthermore, we searched all relevant multimedia report lists for pertinent papers. A medical librarian designed the search strategy, and the search was limited to literature in English. The search results were imported into reference management software (EndNote version 20, New York, NY, USA), and duplicates were removed. The search keywords were selected based on predefined terms and by examining terms in medical rubrics (MeSH). The search strings included combinations of the following terms: “interventional,” “software,” “angio-suite,” “procedural,” and “digital product.”

### Data Abstraction

Seven general practitioners (FBS, RIO, AAM, SAE, ASA, AMS, and ABD) conducted the literature search independently and in duplicate in two stages (first, titles and abstracts, followed by full texts) to identify eligible studies. Any disagreements were analyzed and resolved by discussion, consensus, and when necessary, consultation with three radiology consultants (AMA, MSA, and BFD) who subspecialized in IR. A total of 44 resources were found in the PubMed-database-indexed literature that met our eligibility criteria. We reviewed the definitions, main features, functionality details, and software requirements of various software applications from the available literature, as well as user guides.

## 3. Discussion

**Figure 1:**
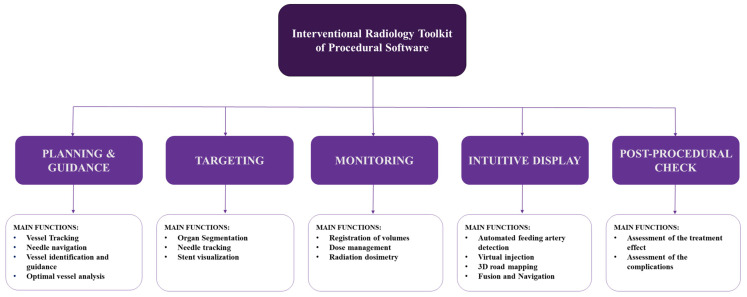
Flow diagram derived from Linte et al. and Floridi et al. [8,9] and algorithmicized by Al-Sharydah et al. to classify the Interventional Radiology toolkit of procedural software.

Linte et al. [8] and Floridi et al. [9] reported that image-guided procedures could be classified on the basis of their implementation steps. An image-guided procedure comprises the following five distinct steps wherein software can be used: preprocedural PLANNING AND GUIDANCE, periprocedural TARGETING, procedural MONITORING, INTUITIVE DISPLAY, and POSTPROCEDURAL CHECK/FOLLOW-UP (Figure 1).

### 3.1. Planning and Guidance

In IR, this step involves the acquisition of preoperative data (typically multimodal images) for target lesion visualization. At this stage, software applications are used for planning vessel tracking, tool navigation, vessel identification and guidance, and optimal vessel analysis. Commonly used software products are listed below.

#### 3.1.1. SmartCT

This image acquisition, visualization, and measurement software was designed for three-dimensional (3D) visualization of the vasculature, soft tissues, and bone structures, as well as for hemorrhages. It assists physicians in identifying pathologies and can, thus, assist in the formulation of appropriate interventional strategies [10,11,12,13]. It enhances 3D imaging by simplifying 3D acquisition, provides clear guidance for popular 3D interventional tools, and ensures swift automatic display of 3D images on a touch-screen module. Furthermore, it facilitates interactions with advanced 3D visualization and measurement tools. SmartCT can be used in interventional facilities with a Philips Interventional X-ray system. According to the Global Medical Device Nomenclature (GMDN), SmartCT is an angiographic X-ray system application software [10,11,12,13].

##### SmartCT Vaso

This high-resolution 3D-neuroimaging technique provides vital data on cerebral vascular structures for vessel assessment in the soft tissue context. It has abundant features, such as the ability to visualize submillimeter vasculature and structures during neuro-procedures, and offers step-by-step guidance for 3D imaging.

SmartCT Vaso can be used at interventional facilities along with a Philips Interventional X-ray system. According to the GMDN, it is an angiographic X-ray system application software [11,12].

##### SmartCT Soft Tissue

**Figure 2:**
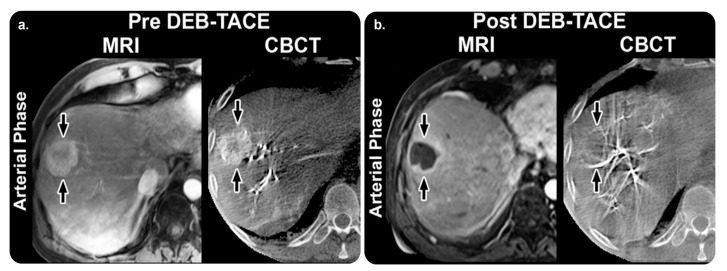
SmartCT software soft tissue technique and its role in management planning. (**a**) Arterial-phase images obtained before TACE. MRI revealing a 75 mm mass in the right lobe “arrow” with 65% enhancement. The mass has a similar enhancement (75%) with CBCT. (**b**) Arterial-phase images obtained after TACE. Mass shows almost no enhancement (5%) with MRI, and the tumor is decreased to 69 mm. With CBCT, the intraprocedural mass enhancement is decreased by 87%, which allows prediction of an objective EASL response at 1 month. Abbreviations: TACE, transarterial chemoembolization; MRI, magnetic resonance imaging; CBCT, cone-beam computed tomography; EASL, European Association for the Study of the Liver. Reprinted from “Intraprocedural C-arm dual-phase cone-beam CT: Can it be used to predict short-term response to TACE with drug-eluting beads in patients with hepatocellular carcinoma?” by Loffroy et al. [14] with written approval from the corresponding author and publisher (Radiology; Copyright 2013).

This X-ray acquisition technique enables computed tomography (CT)-like visualization of soft tissue structures periprocedurally; CT-like images aid in soft tissue and bone structure assessments and stent deployment. This technique also guides cone-beam CT (CBCT) image acquisition in a step-by-step manner. Additionally, it enables table-side access to advanced 3D measurements and efficient interaction with CBCT images on a touch-screen module. It also provides open CBCT protocols for liver procedures. SmartCT Soft Tissue can be used in interventional facilities along with a Philips Interventional X-ray system. According to the GMDN, it is an angiographic X-ray system application software [11,12] (Figure 2).

##### SmartCT Angio

This is a touch-screen-enabled X-ray acquisition technique for table-side 3D visualization of the cerebral, abdominal, cardiac, and peripheral vasculature during a single rotational angiography session. Furthermore, it incorporates interactions with 3D-imaging tools and allows for the selection of two vessel points for a more defined vessel path. SmartCT Angio can be used in interventional facilities along with a Philips Interventional X-ray system. According to the GMDN, it is an angiographic X-ray system application software [11,12].

The available practical evidence is as follows. 

Loffroy et al. [14] conducted a prospective observational study to evaluate whether C-arm dual-phase CBCT helped determine tumor response in 50 targeted hepatocellular carcinomas (HCCs) in 29 patients who underwent transarterial chemoembolization (TACE). Their findings revealed that intraprocedural C-arm dual-phase CBCT could be used to determine HCC response at the 1-month follow-up point.

Higashihara et al. [15] performed a prospective observational study on 30 patients and obtained equivalent findings regarding HCC detection between C-arm CT during TACE and multidetector CT.

Miyayama et al. [16] performed a retrospective observational study on 207 HCCs treated with TACE with digital subtraction angiography (DSA) alone (98 tumors in 70 patients) or with TACE with DSA plus CBCT monitoring (109 tumors in 79 patients). They concluded that, compared with DSA alone, DSA with CBCT improved the technical success of TACE and reduced the local tumor recurrence rates.

#### 3.1.2. AdvantageSim MD

This adaptive virtual simulation software automatically prescribes contours, volumes, and geometric beam placement to enhance the accuracy and velocity of dosimetry planning for high-precision radiotherapy techniques [17]. It incorporates simulation and localization technology and enhances productivity and accuracy. It enables multimodality simulation (CT, magnetic resonance imaging (MRI), and positron emission tomography (PET)), which provides additional data and helps maintain accurate treatment plans on one desktop. It also helps manage innovative 4D CT and 4D PET/CT workflows and achieves pelvic organ segmentation with semi-automated MR. This software achieved Digital Imaging and Communications in Medicine (DICOM)-Radiation Therapy and Integrating Healthcare Enterprise-Radiation Oncology compliance for smooth interoperability. AdvantageSim MD can be used on an Advantage Workstation (AW) 4.6 and an AW Server 2 [17]. Our search revealed no evidence of its practical clinical impact.

#### 3.1.3. AngioCARD

This software utilizes customizable tools to assist referring physicians in generating detailed reports with schematics and images for MR and CT vascular studies. It imports medical images and displays them on a workstation screen. Furthermore, it shows movies and static images in many familiar formats. It also archives reports, cine images, and clinical notes in DICOM and electronic forms. It has a user-friendly interface and provides valuable information (such as acquisition mechanism, stenosis severity, and vascular schematics) in customized reports. AngioCARD can be used with AW 4.2 (or higher) and is compatible with AW postscript printers [18]. Our search revealed no evidence of its practical clinical impact.

#### 3.1.4. AngioViz

**Figure 3:**
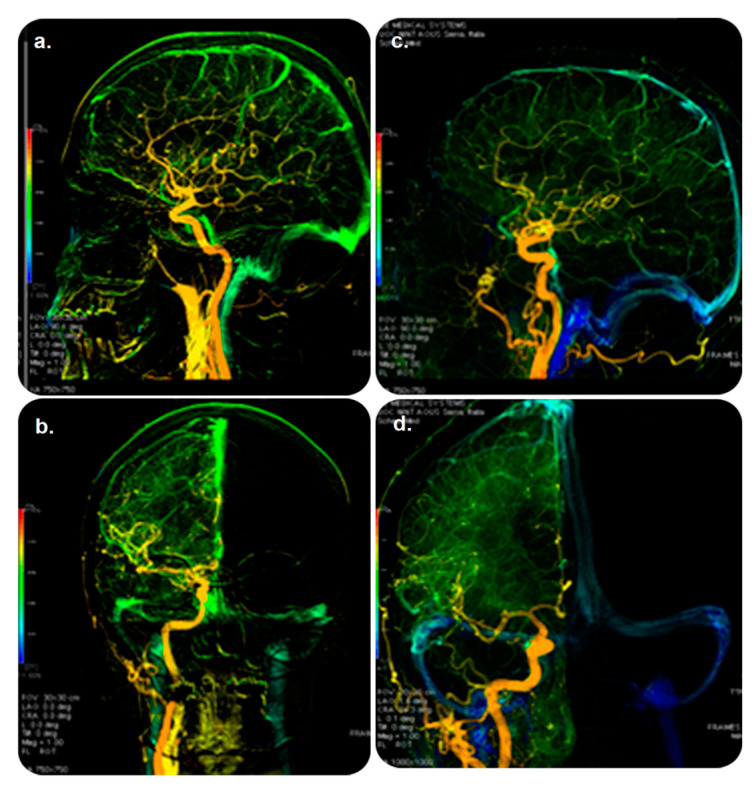
AngioViz software and its role in guiding therapy. DSA examination: (**top**) lateral and (**bottom**) anterior–posterior views of color-coded right carotid artery in controls (**a**,**b**) and patients (**c**,**d**) with multiple sclerosis. Blue color shown only in patients demonstrates prolonged cerebral circulation time. Veins are in green (and not in blue) in the controls (**a**,**b**). Abbreviations: DSA, digital subtraction angiography. Reprinted from “Cerebral circulation time is prolonged and not correlated with EDSS in multiple sclerosis patients: A study using digital subtracted angiography” by Monti et al. [20] with approval from the corresponding author and publisher (PLOS ONE; Copyright 2015 [open access license]).

This software uses multicolored DSA to produce sequenced parametric images of peak opacification simultaneously with peak times to allow users to visualize vascular-flow-related features. It examines a single image and summarizes the major data contained in a DSA time series to recognize vascular flow. Furthermore, AngioViz automatically synchronizes various DSA series for flow comparison and can interpret a complex flow model of several anatomical regions. AngioViz can be used with AW VolumeShare 5 and higher [19]. Our search revealed no evidence of its practical clinical impact (Figure 3).

#### 3.1.5. Autobone and VesselIQ Xpress

This optimized, noninvasive application is used for analyzing vascular anatomy and pathology with rapid tracking of all vasculatures. The designing of appropriate treatment plans based on a set of CT angiographic images ensures no bone segmentation of the head, neck, and other regions. It automatically detects the aorta, iliac vessels, and thrombi and can auto-label vessels. Furthermore, 3D or reformatted vessel images can be analyzed with one click. This software can be used on an AW workstation and AW Server platforms [21]. Our search revealed no evidence of its practical clinical impact.

#### 3.1.6. Hepatic VCAR

**Figure 4:**
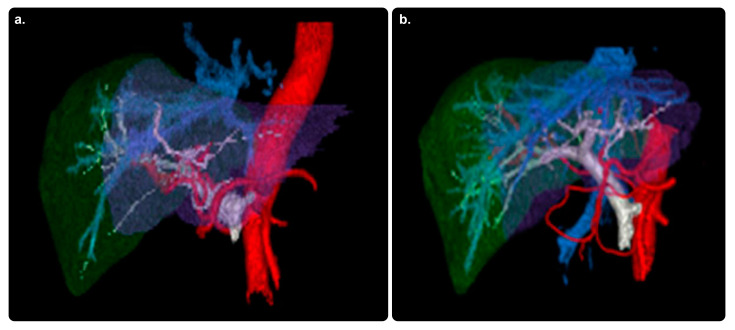
Hepatic VCAR software and its role in therapy planning. Color-enhanced 3D volume-rendered images of livers from two patients (**a**,**b**) with hepatic malignancies. 3D, three-dimensional; red, hepatic arteries arising from the aorta; blue, hepatic veins leading to the inferior vena cava; gray, portal venous system; green, right-lobe liver parenchyma; purple, left-lobe liver parenchyma. Reprinted from “Comparative analysis of three-dimensional volume rendering and maximum intensity projection for preoperative planning in liver cancer” by Ho et al. [23] with written approval from the corresponding author and publisher (European Journal of Radiology Open; Copyright 2020).

This software allows for guided segmentation and assessment of the liver, lesions, and vascular system through the use of an automatic contour tool, enabling a more consistent workflow in interventional planning. Furthermore, it provides an extensive learning algorithm for the segmentation of the liver and its hepatic arteries at different phases. Finally, it measures the tumor burden and facilitates communication using built-in efficient and consistent reporting tools [22]. Our search revealed no evidence of its practical clinical impact (Figure 4).

#### 3.1.7. Innova CT HD and Innova 3D

These next-generation imaging software packages aid physicians in establishing diagnoses and adapting procedural planning and treatment follow-ups by reconstructing 3D volumes from rotational fluoroscopy. They can display high-quality axial, sagittal, coronal, and oblique cross-sections, which in turn enable the generation of images with fewer streak artifacts (without increasing doses) and unification of images for soft tissue visualization, as well as yield high locative resolution for enhanced visualization of micrometric devices. These packages can be used on an AW workstation and AW Server platforms [24,25]. Our search revealed no evidence of their practical clinical impact.

#### 3.1.8. MR VesselIQ Xpress

This software enables flexible vascular anatomy, pathology, and vessel analyses from a set of DICOM-compliant 3D contrast-enhanced MR angiography (MRA) images. It enables measurement, display, batch filming, and archive traits that enable the analysis of selected vessels for directional tortuosity, stenosis, and other anomalies. It helps promote oriented workflows and innovative layouts for smoother vascular MR data analysis, shortens the waiting time for the first clinically relevant image, and provides rapid 3D visualization and access to vessel cross-section and profile images. Furthermore, the software can be used on a personal computer, laptop, or picture-archiving and communication system (PACS)/radiologically isolated syndrome workstation for a smooth workflow. The software runs on AW workstations and server platforms [26]. Our search revealed no evidence of its practical clinical impact.

#### 3.1.9. TEVAR Assist Solutions

This software helps assess cardiovascular and vascular disease through the analyses of 2D and 3D CT angiography (CTA) images obtained from DICOM-compliant CT scans. It supports physicians in vessel analysis, pre- and post-stent planning, and vessel tortuosity visualization. It can be used to analyze CTA data with zero-click bone removal and aorta tracking, to perform key anatomy measurements, and to superimpose 3D CTA data over live fluoroscopic images. Furthermore, it helps achieve efficient procedure times and doses by enabling direct registration in table-side CT models with only two fluoroscopy views; it also facilitates registration of real-time 3D to C-arm and table movements, view range, and source-to-image distance. It allows smooth follow-up with semi-automatic sizing of the thrombus. This software can be used with Volume Viewer AW VolumeShare 4 In-room AW monitors compliant with CT scanners and PACS stations [27]. Our search revealed no evidence of its practical clinical impact (Table 1).

### 3.2. Targeting

In an IR procedure, this step involves localization and tracking of the position of surgical tools and therapeutic devices. At this stage, software applications are used for organ segmentation, tools, vessel tracking, and stent deployment visualization. The applications are listed below.

#### 3.2.1. StentBoost Live

**Figure 5:**
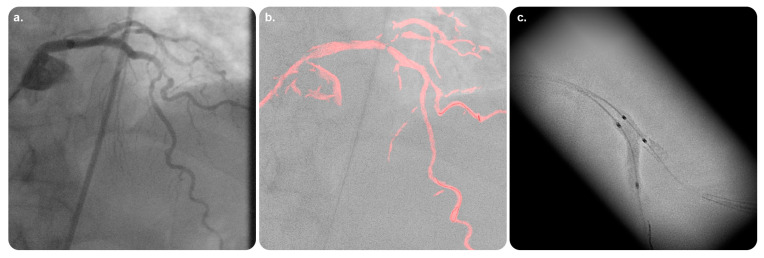
StentBoost Live software and its role in the delivery of targeted therapy. (**a**) Standard static road map with contrast media. (**b**) Coronary road map fused with live fluoroscopy image showing both wires exactly superimposed on the coronaries. (**c**) The software algorithm automatically searches for proximal and distal balloon markers and displays an enlarged and enhanced image of the area around them to insure precise balloon-mounted stent deployment. Reprinted from “On the road: First-in-man bifurcation percutaneous coronary intervention with the use of a dynamic coronary road map and StentBoost Live imaging system” by Dannenberg et al. [32] with written approval from the corresponding author and publisher (International Journal of Cardiology; Copyright 2016).

**Figure 6:**
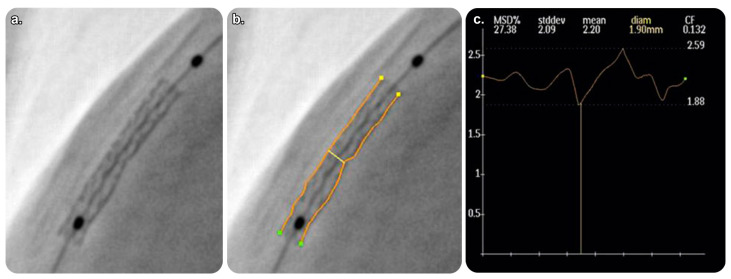
Quantitative analysis of StentBoost software images. (**a**) Enlarged and enhanced StentBoost image. (**b**) Manual tracing of longitudinal stent edges to avoid inadequate stent expansion. (**c**) Minimum stent diameter determined automatically. Reprinted from “Assessment of optimum stent deployment by stent boost imaging: Comparison with intravascular ultrasound” by Tanaka et al. [33] with written approval from the corresponding author and publisher (Heart and Vessels; Copyright 2013).

This live-imaging medical device assists physicians in placing and deploying stents by providing an inclusive visualization of stents in coronary vessels. It allows for procedural efficiency with enhanced visualization during intracoronary device movement and can be consistently integrated into standard-of-care workflows for optimized percutaneous coronary intervention (PCI). StentBoost Live can be used simultaneously with Philips Interventional X-ray software. A PACS system must be operated when using StentBoost Live [28,29,30,31] (Figure 5 and Figure 6).

##### StentBoost Mobile

This is the first dedicated stent-imaging software for mobile C-arm segments. It enables enhanced stent visualization during deployment for better decisionmaking during endovascular treatment and other vascular procedures. It also expands the assessment potential, saves time and money, and supports various vascular interventions [28,29,30,31] (Figure 5 and Figure 6).

The available practical evidence is as follows.

Tanaka et al. [33] conducted a prospective observational study on 68 coronary arteries in 60 patients to compare StentBoost imaging and intravascular ultrasound (IVUS) imaging; they concluded that StentBoost had a relatively low sensitivity for stent placement. However, its specificity was high enough for use in monitoring after stent placement at centers where IVUS was not used.

Cura et al. [34] conducted a prospective observational study on 38 patients to compare the functionality of StentBoost and IVUS during stent placement. They identified a good correlation and agreement between the two as complementary tools, with the minimal stent diameter measured using StentBoost.

#### 3.2.2. XperGuide

This software enables specific, live, 3D needle guidance. It can be used for percutaneous interventions, such as lung and abdominal biopsies, endoleak treatment, vertebroplasties, kyphoplasties, neurobiopsies in neoplastic cranial disease, percutaneous rhizotomy in trigeminal neuralgia, liver biopsies and ablations, and drainage of abdominal fluid collections. Needle navigation technology with CBCT can accurately reach lesions smaller than 1 cm. XperGuide also supports biopsy procedures and drainage during radiofrequency ablations, reduces needle-repositioning time (compared with conventional CT), and is associated with a 29% lower skin dose (compared with conventional CT). XperGuide and XperCT Dual require installation; therefore, they must be used in an interventional workspot [34,35].

##### XperGuide Ablation

This interactive software functions as a live needle guidance tool. The current version performs successful tumor ablations while overriding compromising adjacent tissue; thus, it requires data on the tumor size, the needle’s accurate ablation area, and the correct path to the target. Additionally, it offers customizable isotherm imaging for radiofrequency, microwave, and cryoablation procedures. Furthermore, the software updates interactively during the ablation procedure to ensure ideal tumor coverage [36]. Our search revealed no evidence of its practical clinical impact.

#### 3.2.3. EmboGuide

**Figure 7:**
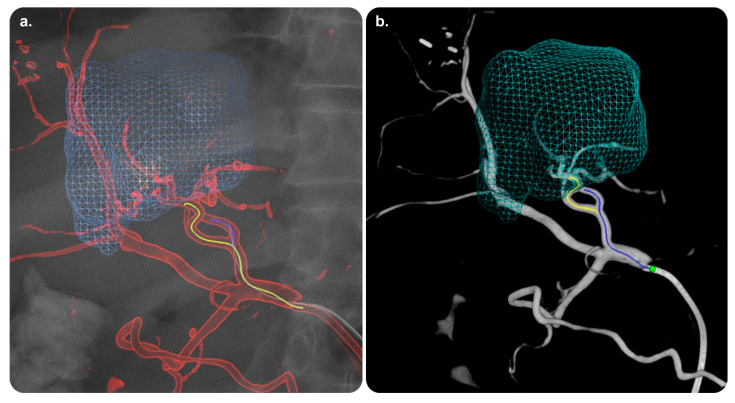
EmboGuide software and its role in the delivery of targeted therapy. (**a**) Feeding arteries superimposed on a live fluoroscopic image during selective catheterization. (**b**) 3D dynamic roadmap displayed on fluoroscopy after dual-phase CBCT enabling target volume segmentation, automatic feeding vessel detection, and selective microcatheterization of feeding vessels. Reprinted from “First human experience with directly image-able iodinated embolization microbeads” by Levy et al. [39] with written approval from the corresponding author and publisher (CardioVascular and Interventional Radiology; Copyright 2016).

EmboGuide with XperCT Dual provides workflow-based embolization guidance and automatically detects and treats tumors and vessel suppliers to multiple lesions. Compared with DSA, EmboGuide can detect 50% more HCC feeders [37,38]. Furthermore, it overlays 3D reconstructions on X-ray images to facilitate navigation to an embolization target and maximize the performance of TACE procedures [37,38]. Our search revealed no evidence of its practical clinical impact (Figure 7).

#### 3.2.4. Vision 2

This 3D live guidance software operates by preparing 3D datasets during real-time fluoroscopy to facilitate the localization and guidance of catheters, coils, and other devices during IR procedures. The current version offers a table-side intuitive interface and accurate 3D anatomy registration with a bi-view mode that eases registration with the radiation dose savings mode. Version 2 can be used with AW at AW 4.7 (VolumeShare 7) or higher and dedicated Volume Viewer products. Furthermore, this software requires one of the following X-ray systems: InnovaTM IGS 5, InnovaTM IGS 6, DiscoveryTM IGS 7, DiscoveryTM IGS 7 OR, AlliaTM IGS 5, AlliaTM IGS 7, or AlliaTM IGS 7 OR [40].

##### TrackVision 2

TrackVision 2 provides live 3D needle guidance during IR procedures. The loading of real-time 3D datasets and overlay enables needle advancement through a planned trajectory overlaid on live fluoroscopy and highlights any deviations from the desired path. It supports various trajectories and facilitates the registration of real-time 3D trajectories to C-arm and table movements. Furthermore, it displays patient motion and bone anatomy overlay with table-side correction. Thereafter, it smoothly sends a bulls-eye view angle to the gantry. TrackVision 2 can be used with Volume Viewer and AW VolumeShare 4 or higher [41]. Our search revealed no evidence of its practical clinical impact (Table 2).

### 3.3. Monitoring

In an IR procedure, this step involves the registration of localized volume and confirmation of the device position on the basis of preoperative data. At this stage, software applications are used for periprocedural radiation dosimetry as well. Commonly used applications are listed below.

#### 3.3.1. AneurysmFlow

**Figure 8:**
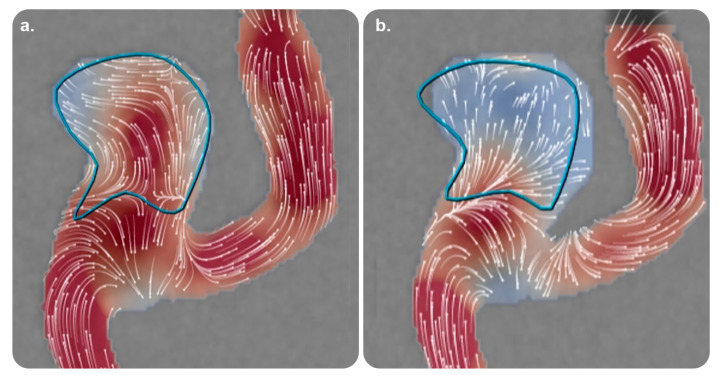
AneurysmFlow software and its role in monitoring therapy. Volume reconstruction images utilizing high-frequency digital subtraction angiography of two different Medina^®^ Embolization Device configurations (a and b) after implantation in the same aneurysm model. (**a**) Incomplete neck coverage corresponding to moderate flow velocity reduction (41%). (**b**) A different configuration showing better coverage of the neck corresponding to a higher degree of intra-aneurysmal flow velocity reduction (73%). Reprinted from “Intra-aneurysmal flow disruption after implantation of the Medina^®^ embolization device depends on aneurysm neck coverage” by Frölich et al. [47] with permission from the corresponding author and the publisher (PLOS ONE; Copyright 2018 [with an open access license]).

AneurysmFlow is a quantification tool for IR procedures and is also the first interventional software to depend on angiography for visualizing blood flow patterns in a cerebral aneurysm and its parent artery before and after flow diverter deployment [42]. It provides new data (with the mean aneurysm flow amplitude ratio) and enhances clinical decision making. AneurysmFlow can be used with a Philips Interventional X-ray system and 3D rotational angiography data [43,44,45,46] (Figure 8).

The available practical evidence is as follows.

Cancelliere et al. [48] conducted a trial on an in vitro experimental setup comprising four patient-specific silicone models (eight devices and stents) and reported that AneurysmFlow was useful for determining the mean aneurysm flow amplitude and intra-aneurysmal flow changes in all the cases.

Sforza et al. [43] performed a literature review and concluded that the physiological blood flow parameters that regulate aneurysm morphology and natural history are poorly understood. It is necessary to model intra-aneurysmal hemodynamics using realistic aneurysm geometries because aneurysm geometry is one of the most critical factors for determining the aneurysm flow patterns that influence aneurysm progression.

#### 3.3.2. Volume Viewer

This is a premium advanced visualization and image-processing platform that provides a rich 3D-image-processing toolset to create and display required views with little user input. It helps streamline interpretation and reporting by providing visualization tools with a minimal number of clicks. It has a modern user interface with a vast viewing space for clinical images, as well as intuitive tools for the annotation, measurement, and segmentation of structures of interest [49,50]. The toolbar is customizable; multiple comparable views can be derived from different modalities, and the platform can be used for 3D reformatting, maximum intensity projection (MIP)/multiplanar reformation or reconstruction, and high-resolution volume rendering. The platform enables inclusive and easy tracking of vascular structures. Furthermore, it provides an integrative summary schedule with measurements reflected in images and a flexible layout that can be used on a dual-monitor system in landscape and portrait orientations. Volume Viewer can be used in a number of international languages and runs on the following: AW Server 3.1 and above, dual-monitor resolutions up to 2MP (1600 × 1200) or a single 3MP (1536 × 2048), AW VolumeShare7 Workstation, and CentricityTM Universal Viewer3 [49,50]. Our search revealed no evidence of its practical clinical impact.

#### 3.3.3. Volume Viewer Innova

This software efficiently processes radiographic, CT, and MR 3D models to assist users during clinical practice. The current version offers a multi-oblique mode for planning needle trajectories in the trajectory-planning protocol. It automatically segments the left atrium on Innova3D/CT and the left cavity on CT [49,50]. Furthermore, it has a Wizard panel that can be used to enhance multivolume segmentation workflows. It enables the periprocedural positioning of landmark points and planning lines, as well as connectivity to the interventional suite. This application can be used with single and dual color monitors with AW Server 3.1 (or higher) and with dual monitors with AW version 4.7 (or higher) in the portrait and landscape orientations [49,50]. Our search strategy revealed no evidence of its practical clinical impact (Table 3).

### 3.4. Intuitive Display

In an IR procedure, this step involves virtualizing the position of a tool concerning medically necessary structures visible in preoperative images. At this stage, software applications are used for automated feeding artery detection, virtual injection, 3D roadmapping, registration and fusion, navigation, virtual injection, and embolization. Software commonly used at this stage are listed below.

#### 3.4.1. SmartCT Roadmap

This interactive software provides live 3D segmented images processed with SmartCT Angio or SmartCT Soft Tissue to emphasize the targeted vessels and lesions. It has abundant features and can be accessed at the table via a touch screen. It provides dynamic 3D guidance for various applications, which facilitates catheter navigation through the synthetic visualization of vessel structures. Furthermore, it adjusts to emerging changes. Compared with standard 3D images, images from this software have enhanced transparency and visibility. SmartCT Roadmap can be used in an interventional workspot with a Philips Interventional X-ray system. According to the GMDN, it is an angiographic X-ray system application software [10,11,12,13]. Our search revealed no evidence of its practical clinical impact.

#### 3.4.2. VesselNavigator

**Figure 9:**
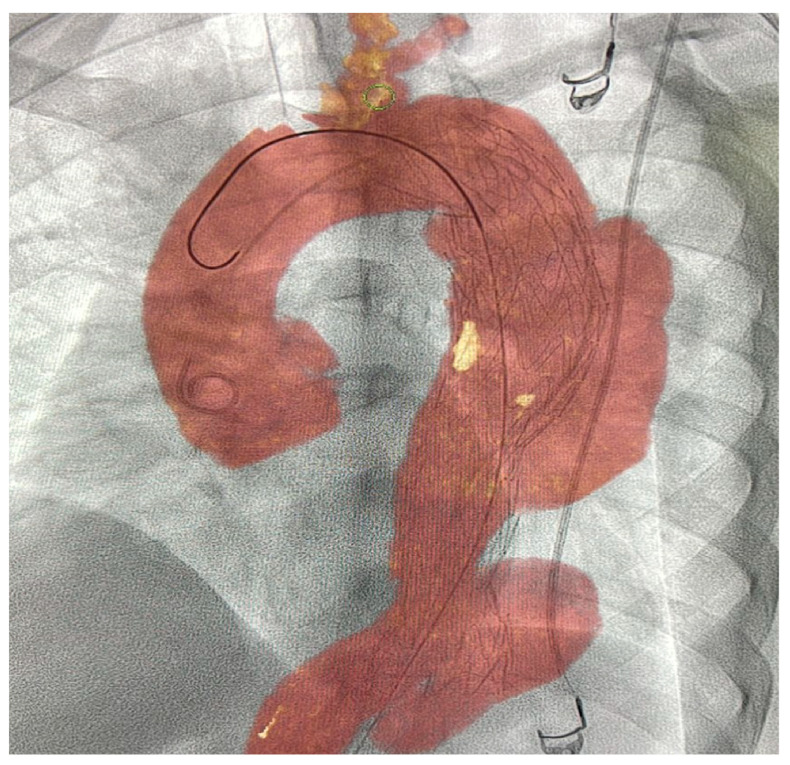
VesselNavigator software and its role in intuitive display. Superimposed image on a live fluoroscopic image utilizing VesselNavigator software during EVAR planning for a patient with Stanford type-B aortic dissection. Stent deployment during endovascular EVAR procedure with software monitoring for proper placement along branching arteries. EVAR was performed uneventfully, and the flow was restored into branching arteries. Abbreviations: EVAR, endovascular aneurysm repair. (Original copyrights).

This 3D image fusion and navigating device can be used to overlay a 3D dataset on live fluoroscopy images of the same anatomy during IR procedures [51]. Using 3D image fusion technology, it facilitates advanced endovascular procedures [52]. It can be used to navigate complex vessels and structures and improve clinical outcomes. Furthermore, it helps minimize contrast-enhanced runs, and a pre-acquired CTA or MRA database provides real-time 3D vascular anatomical information on a live X-ray image. VesselNavigator can be used in combination with a Philips Interventional X-ray system [53,54] (Figure 9).

The available practical evidence is as follows.

De Ruiter et al. [55] conducted a meta-analysis of 27 articles including 3444 patients to examine the total duration of fluoroscopy and radiation exposure dose during endovascular aortic repairs by mobile, fixed, or fixed C-arms with 3D image fusion. The findings asserted that associating fixed C-arms with 3D image fusion techniques in complex cases was sufficient for compensating for higher radiation doses measured while utilizing a fixed C-arm. 

Stangenberg et al. [56] in a retrospective observational study subjected 75 patients to aorto-bi-iliac endovascular aneurysm repair to investigate the potency of VesselNavigator in facilitating accurate and swift procedures and reducing the radiation exposure and contrast agent dose. They concluded that VesselNavigator produced less radiation exposure for patients and interventionists than conventional devices. Furthermore, it maintaineed a limited amount of required contrast agent and shortens the overall procedure length. 

#### 3.4.3. HeartNavigator

**Figure 10:**
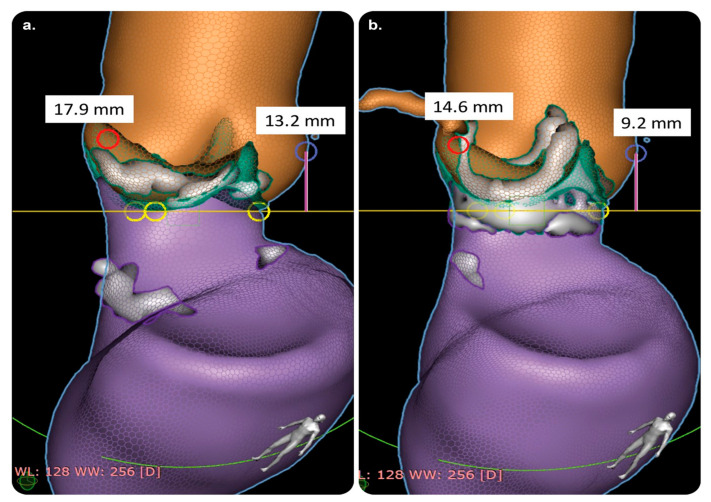
HeartNavigator software and its role in intuitive display. CT assessment of coronary heights before and after aortic valve replacement with implantation of the ‘RD-AV EDWARDS INTUITY’ system. (**a**) Preoperative RCAA and LCAA heights (red and blue circles, respectively; distance from the annular plane to the left coronary ostium). (**b**) Postoperative RCAA and LCAA heights (red and blue circles, respectively; distance from the annular plane to the right coronary ostium). Abbreviations: CT, computed tomography; LCAA: left coronary artery-to-annulus; RCAA: right coronary artery-to-annulus. Reprinted from “Effect of conventional and rapid-deployment aortic valve replacement on the distance from the aortic annulus to coronary arteries” by Coti et al. [60] with written approval from the corresponding author and publisher (Interactive CardioVascular and Thoracic Surgery; Copyright 2021).

This interactive planning software aids cardiac surgeons and interventionalists in insightful planning and determination of the distribution of calcifications. It enables the treatment of structural heart diseases with minimally invasive IR techniques. It can be used to create a volume-rendered 3D heart image for transcatheter aortic valve replacement (TAVR), transcatheter aortic valve implantation (TAVI), and other challenging structural procedures. It automatically secures the entire heart for visualizing the relevant anatomical structures and landmarks. It also simplifies the planning, measurement, and device selection for the ideal X-ray display angles. HeartNavigator offers live image guidance to support periprocedural device adjustments. It can be used in combination with a Philips Interventional X-ray system [57,58,59] (Figure 10).

The available practical evidence is as follows. 

Kočka et al. [61] performed a prospective observational study on 128 consecutive patients with native aortic valve stenosis who underwent TAVI. They compared automated CT analysis using HeartNavigator with standard, manual CT analysis and found that HeartNavigator was a promising and effective technology. The differences in the aortic annulus dimensions were small and similar to the variability observed in manual CT analyses. The automated prediction of optimal fluoroscopic viewing angles was found to be accurate. Moreover, the automated analysis was faster than manual CT analysis; the recommended fluoroscopic viewing angles for TAVI were almost identical between the two modalities.

Vaitkus et al. [62] performed a prospective observational study to compare the predictive performances of HeartNavigator and conventional CT for paravalvular leaks in 56 patients who underwent TAVR. They found that, comparable with conventional CT, HeartNavigator was a more reliable tool, allowed accurate assessments, and aided in prosthetic size selection.

Coti et al. [60] performed a retrospective observational study on 112 patients who underwent surgical aortic valve replacement with either a rapid-deployment aortic bioprosthesis (EDWARDS INTUITY Elite Valve) or other standard, sutured biological valves. They assessed coronary height using HeartNavigator.

#### 3.4.4. EP Navigator

**Figure 11:**
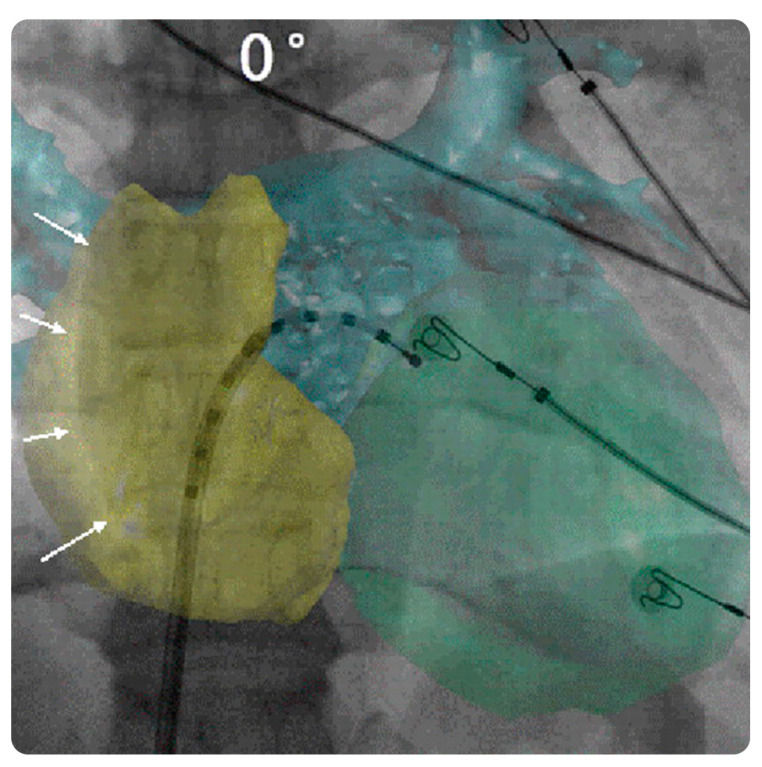
EPNavigator software and its role in intuitive display. CT registration based on heart contours (including reconstruction of both the atria and left ventricle). Antero-posterior fluoroscopic view with CT overlay. A decapolar catheter is inserted into the left atrium through a trans-septal sheath. The CT volume overlay is first aligned using the right lateral atrial contour (white arrows). Abbreviations: CT, computed tomography. Reprinted from “Computed tomography–fluoroscopy overlay evaluation during catheter ablation of left atrial arrhythmia” by Knecht et al. [66] with written approval from the corresponding author and publisher (EP Europace; Copyright 2008).

This catheter navigation software uses pre-interventional CT or CTA images as a reference to generate a 3D image of the heart and registers 3D images with live fluoroscopy to facilitate catheter navigation during atrial fibrillation (AF) ablation [63]. These composite images can be displayed as reference images on a monitor in an examination room during navigation. Furthermore, the 3D model obtained from CT, MRI, or X-ray angiography can be used to show a segmented heart structure in detailed 3D images with enhanced anatomy; these can be overlaid onto 2D live fluoroscopy to enhance complex procedures. EP Navigator can be used with a compatible Philips Interventional X-ray system [64,65] (Figure 11).

The available practical evidence is as follows. 

Knecht et al. [66] performed a prospective observational study on 56 patients with symptomatic AF who underwent contrast CT of the left atrial and pulmonary veins prior to ablation. They demonstrated the feasibility of the EP Navigator and reported that it allowed accurate left atrial mapping and ablation and facilitated transseptal puncture.

Fujita et al. [67] performed a prospective observational study on 114 patients who underwent catheter ablation for AF and compared the feasibility, efficacy, and safety of intraprocedural reconstructions of 3D cardiac images between 3D rotational angiography (3D-ATG) and preprocedural CT/MRI. The effective dose (particularly the surface radiation dosage) for 3D-ATG was significantly lower than that for CT. Furthermore, the anatomical accuracy was comparable between 3D-ATG and CT.

#### 3.4.5. EchoNavigator

**Figure 12:**
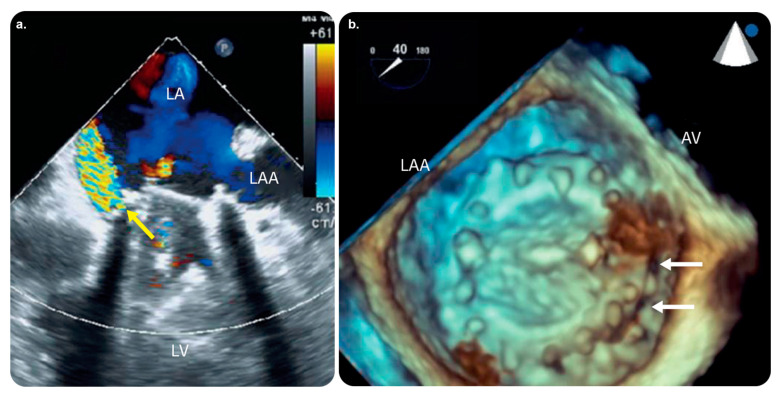
EchoNavigator software and its role in intuitive display. Preprocedural TEE. (**a**) 2D TEE color Doppler image showing severe paravalvular leakage (yellow arrow). (**b**) 3D TEE image showing dark holes between the prosthetic mitral valve and mitral annulus (white arrows) localized at the medial side of the anterior mitral annulus near the aortic valve. Abbreviations: AV, aortic valve; 3D, three-dimensional; 2D, two-dimensional; LA, left atrium; LAA, left atrial appendage; LV, left ventricle; TEE, transesophageal echocardiography. Reprinted from “Three-dimensional EchoNavigator system guided transcatheter closure of paravalvular leakage” by Kim et al. [70] with permission from the corresponding author and the publisher (Journal of Cardiovascular Imaging; Copyright 2019 [open access license]).

This live echo and X-ray fusion software aids interventionalists and surgeons who depend on live 3D transesophageal echocardiography (TEE) and live radiography for guidance during procedures for structural heart diseases. It imports live echo tissue information directly from fluoroscopic imaging and uses X-rays to automatically reflect the markers on soft tissue structures within the echo image. Furthermore, it automatically aligns with the C-arm orientation to interpret the images. The current version (3.0) is designed for use alongside one of the following X-ray systems: Azurion release 1.× to 2.× or Allura Xper release 7.6.× to 8.2.× (including the OR table series). EchoNavigator release 3.0 is also compatible with the following ultrasound systems: EPIQ CVxi Ultrasound devices (release 3.0) and later versions of x8-2t Echo probes [68,69,70,71] (Figure 12).

The available practical evidence is as follows. 

Jone et al. [72] performed a prospective observational study to determine the usefulness of fused echocardiographic/X-ray fluoroscopic imaging (FEX; performed using EchoNavigator) in 25 pediatric patients with congenital heart disease (CHD) who underwent catheterization. They found that FEX reduced the fluoroscopy time and radiation exposure in arterial septal defect closure procedures.

Jungen et al. [73] performed a prospective observational study to investigate whether the automated real-time integration of 2D-/3D-TEE and fluoroscopy imaging in percutaneous left atrial appendage closure decreased radiation exposure to the patients and medical staff.

Ternacle et al. [74] concluded that EchoNavigator improved safety and facilitated communication between interventional team members.

Hadeed et al. [75] performed a prospective observational study on 51 pediatric patients with CHD who underwent TEE-guided catheterization. Image fusion was performed using EchoNavigator in all the cases. They concluded that EchoNavigator was a useful modality in terms of safety and aided interventionists in better recognizing the anatomy.

#### 3.4.6. FlightPlan for EVAR

This software is designed to simplify the sizing and planning for EVAR on MRA or CTA. It aids in visualizing vascular anatomy, performing key anatomical measurements, selecting a treatment strategy, measuring endograft size, and saving key information that can be used during an intervention [76]. Moreover, the current software enables zero-click bone removal and CTA tracking of abdominal vessels. It expedites key aorta-sizing measurements and can analyze the endograft-sealing zones. Furthermore, FlightPlan generates and saves the volume, C-arm angulations, and vessel ostia contours to be used periprocedurally. The current version can be used with AW VolumeShare 7 workstations (or higher), Volume Viewer, Volume Viewer Innova, VesselIQ Xpress, AutoBone Xpress, and MR VesselIQ Xpress [76]. Our search revealed no evidence of its practical clinical impact.

#### 3.4.7. FlightPlan for Liver

**Figure 13:**
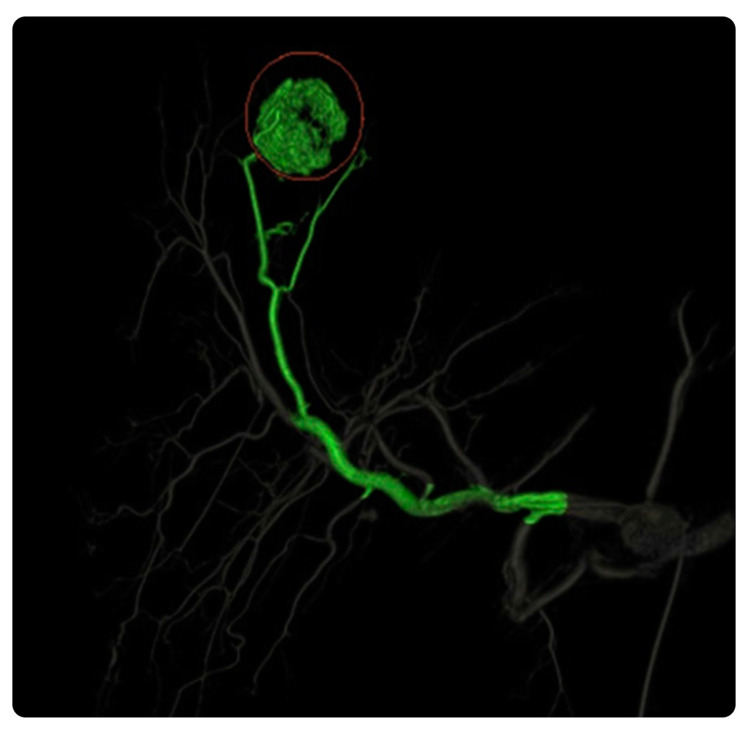
FlightPlan for Liver software and its role in intuitive display. FlightPlan for Liver enables the isolation of the relevant arterial vascular supply, automated vessel detection, and color coding (green) of arteries supplying the designated hypervascular region of interest. Reprinted from “Assessment of automated cone-beam CT vessel identification software during transarterial hepatic embolization: Radiation dose, contrast medium volume, processing time, and operator perspectives compared to digital subtraction angiography” by Durack et al. [78], Copyright 2018, with written approval from the corresponding author and publisher (Clinical Radiology. Copyright 2018).

This postprocessing liver embolization device can detect liver tumor-feeding vessels and vessels traveling to the tumor vicinity with 95% sensitivity. It can aid physicians in determining the hepatic arterial anatomy during embolization procedures by highlighting hypervascular hepatic lesions. Similar to data on hypervascular tumors, hepatic vasculature data can be easily extracted by clicking on the catheter tip’s location with the target tool [77] (Figure 13).

The available practical evidence is as follows. 

Joo et al. [79] conducted a retrospective observational study to compare the efficiencies of FlightPlan for Liver and DSA in 50 patients with 80 HCC nodules. They concluded that FlightPlan for Liver was more practical than DSA, particularly for detecting small HCCs and small feeding arteries, which are often challenging to detect.

Iwazawa et al. [80] conducted a prospective observational study to evaluate the efficiency of FlightPlan for Liver in identifying the vessels responsible for arterial bleeding during endovascular embolization in five patients with visceral arterial hemorrhages; they found that, due to the accurate, rapid identification of damaged vessels, this software was useful for guiding endovascular embolization, even for visceral arterial hemorrhages.

Ronot et al. [81] conducted a multicenter retrospective observational study to compare the abilities of arterial cone-beam CT and FlightPlan of identifying tumor-feeding arteries in 45 patients with 66 hypervascular HCCs who underwent conventional chemoembolization. They concluded that using the software ensured precise and fast identification of the tumor-feeding vessels.

Durack et al. [78] conducted a unicentral retrospective observational study to compare arterial CBCT using FlightPlan and conventional DSA during TACE in 34 consecutive patients with unresectable hypervascular primary or secondary liver tumors who underwent selective bland or drug-eluting bead TACE. The software was found to have a higher sensitivity for detecting tumor feeder vessels with a reasonable radiation dose and procedural time.

#### 3.4.8. Advantage 4D

This noninvasive image reconstruction CT software can accurately analyze respiratory-induced motion. It illustrates the appearance of anatomical objects in motion, reduces structural deformation, and highlights the dynamic range of motion for precise radiotherapy assessment. It determines the extent and direction of motion and allows the use of either standard or gated therapy. The current version has abundant features. It automatically classifies 4D image data into multiple bins according to the respiratory phase. This enables the best phase range of the anatomy to be viewed in movie-looped motion with 3D or 4D image display and an acquired resolution of up to a 512 × 512 matrix. Advantage 4D can be used to examine the motion profiles created by a vendor device. It also generates multiple-phase series for 2D, 3D, and 4D visualization and creates MIP, Ave-IP, and Min-IP series for more efficient standard or gated therapy delivery. The software is supported on both AW workstation and AW Server platforms [82]. Our search revealed no evidence of its practical clinical impact.

#### 3.4.9. Embo ASSIST with Virtual Injection

This 3D visualization software assists clinicians with dynamically performing embolization procedures by simulating embolization with virtual injection (powered by Edison). It can split the vasculature from cone-beam CT acquisition with one click. Furthermore, it assists clinicians in visualizing and tracking the path of potential injections, thereby supporting the embolization strategy. It also increases 3D live-imaging overlay of vessels for clearer guidance. The application operates on AW workstations with Volume Viewer, Volume Viewer Innova, Vision 2, VesselIQ Xpress, or AutoBone Xpress [83]. Our search revealed no evidence of its practical clinical impact.

#### 3.4.10. EVARVision

This software dynamically integrates 2D X-ray images and 3D models from multiple modalities (CT, MR, and 3D CBCT) in real time to achieve the accurate localization and guidance of catheters, endografts, and other devices during endovascular aortic interventions. It can be used for live-reinforced image guidance to enhance operator comfort. Moreover, it offers a table-side intuitive interface, accurate 3D anatomy registration with a bi-view mode (which eases registration), and a radiation dose-saving mode during registration. It can be used with AW 4.7 (VolumeShare 7) or higher and dedicated Volume Viewer products. Furthermore, one of the following X-ray systems can also be used: InnovaTM IGS 5, InnovaTM IGS 6, DiscoveryTM IGS 7, DiscoveryTM IGS 7 OR, AlliaTM IGS 5, AlliaTM IGS 7, or AlliaTM IGS 7 OR [84]. Our search strategy revealed no evidence of its practical clinical impact.

#### 3.4.11. Stereo 3D

This is a product combining Vision 2, EVARVision, and TrackVision 2. It permits users to reconstruct 3D objects from two spatially sectioned fluoroscopic acquisitions, which assists physicians in localizing devices during 3D anatomy visualization without CBCT acquisition. The reconstructed 3D objects are composed of needles, points, and line segments. The current version provides a table-side intuitive user interface and a radiation dose-saving mode. Furthermore, it enables physicians to accurately and rapidly localize needles and reconstruct markers using 3D technology. Stereo 3D can be used with AW 4.7 (VolumeShare 7) or higher and dedicated Volume Viewer products. InnovaTM X-ray systems can also be used [85]. Our search revealed no evidence of its practical clinical impact. The main features of the aforementioned intuitive display software are summarized in Table 4.

### 3.5. Postprocedural Check

In an IR procedure, this step involves assessment of the treatment effect and complications. Software commonly used at this stage are listed below.

#### 3.5.1. SmartPerfusion

**Figure 14:**
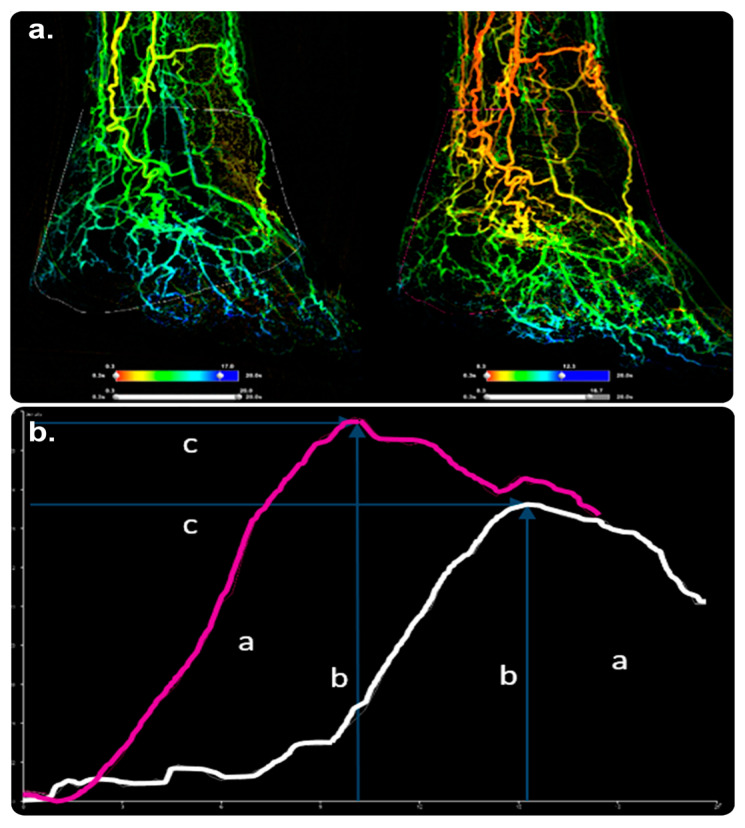
SmartPerfusion software and its role in postprocedural checking. (**a**) 2D reconstituted perfusion images of the foot in anterior–posterior views before and after PTA of the tibioperoneal trunk. ((**a**), right image) shows higher blood supply in the foot after treatment. (**b**) A significant increase in contrast passage, as demonstrated by a larger AuC (a), higher peak density (b), and shorter time to peak (c). SmartPerfusion demonstrated a significant improvement in foot perfusion, unlike DSA, which failed to show a convincing treatment impact. Abbreviations: AuC, area under the curve; DSA, digital subtraction angiography; PTA, percutaneous transluminal angioplasty. (Original copyrights).

This image-intensified fluoroscopic X-ray software provides color-coded DSA images; visualizes manifold functional parameters; and compares preprocedural, periprocedural, and postprocedural color-coded images. It utilizes perfusion-imaging technology for locating the treatment endpoint. It supports physicians in determining the outcomes of perfusion procedures through instant perfusion parameter changes. It also allows standardized pre- and post-comparisons through automated guided positioning. SmartPerfusion can be used with a compatible Philips Interventional X-ray system [86,87] (Figure 14). Our search strategy revealed no evidence of its practical clinical impact.

#### 3.5.2. Innova 3D

This 3D-imaging software aids physicians in establishing diagnoses, visualizing complex vasculature, and adapting interventional procedures and treatment follow-ups by reconstructing 3D volumes using rotational fluoroscopy. With full-resolution volume reconstruction and a streamlined workflow displayed on a monitor, Innova 3D can be used to visualize axial, sagittal, coronal, and oblique cross-sections [24]. Our search revealed no evidence of its practical clinical impact.

#### 3.5.3. Innova CT HD

This next-generation 3D-imaging software aids physicians in establishing diagnoses and adapting surgical planning, interventional procedures, and treatment follow-ups by reconstructing 3D volumes from rotational fluoroscopy. The cross-sections’ quality helps limit streak artifacts without increasing the dose and unifies images for soft tissue visualization. Furthermore, the software has high locative resolution for the enhanced visualization of micrometric devices. Innova CT HD can be used with an AW workstation and AW Server platforms [25]. Our search revealed no evidence of its practical clinical impact. The main features of the aforementioned postprocedural check software are summarized in Table 5.

## 4. Conclusions

This review represents a first step toward establishing a standard classification system that guides interventionalists in choosing a software toolkit for clinical decision making in periprocedural phases and post-therapy assessments within angiographic suites. It defined the purpose, functionality, and implementation requirements of 36 software packages. Classification of these software packages revealed that the majority are used in the intuitive procedural display and preprocedural-planning stages. Most studies have examined the clinical uses of software packages during the intuitive display stage. Studies on the use of software in other stages, as well as on those that can combine complex functions from different stages, have not yet been reported. This review summarized the current knowledge on commonly used software and identified gaps that can be used to prioritize future research (such as prospective comparative clinical trials) and improve patient care.

## Figures and Tables

**Table 1:** Summary of commonly available (Planning and Guidance) procedural software used in interventional therapies.

**Category**	**Software**	**Main Features**
PLANNING and GUIDANCE	SmartCT	Visualizes 3D vasculature Interacts with advanced 3D visualization and measurement tools
	SmartCT Vaso	3D neuroimaging software Visualizes structures and submillimeter vasculature Comprehensively guides 3D-imaging acquisition
	SmartCT Soft Tissue	Enables CT-like visualization Soft tissue and bone structure assessment for stent deployment Simple cone-beam tomography acquisition
	SmartCT Angio	3D visualization by X-ray acquisition Determines vessel paths through two selected points
	AdvantageSim MD	Prescribes contour, volume, and geometric beam placement Dosimetry planning for high-precision radiotherapy Effective multimodality simulation Semi-automated MR-led pelvic organ segmentation
	AngioCARD	Generates meticulous schematics and image reports for MR and CT vascular studies Archives reports, cine images, and clinical notes in DICOM and electronic forms
	AngioViz	Generates sequenced parametric images with peak opacity Simplifies flow models of several anatomical regions
	Autobone and VesselIQ Xpress	Analyzes vascular anatomy and enables rapid vascular pathology tracking No bone segmentation by CT angiography Effortless 3D analyses and vessel image reformatting
	Hepatic VCAR	Oriented liver segmentation and assessment Disparate algorithm for liver and hepatic artery segmentation
	Innova CT HD and Innova 3D	Rotational fluoroscopy for 3D volume reconstruction Provides high-quality axial, sagittal, coronal, and oblique cross-sections
	MR VesselIQ Xpress	Flexible vascular anatomy, pathology, and vessel analyses Rapid 3D visualization and access to vessel cross-section
	TEVAR Assist Solutions	2D and 3D CT angiography analyses Zero-click bone removal, aorta tracking, and key anatomy measurements 3D CTA data overlaid on live fluoroscopic images

**Table 2:** Summary of commonly available (TARGETING) procedural software used in interventional therapies.

**Category**	**Software**	**Main Features**
**TARGETING**	StentBoost Live	Inclusive stent visualization in coronary vessels
	StentBoost Mobile	Enhanced stent visualization during deployment Facilitates proper decision making during endovascular treatment
	XperGuide	Specific, live 3D needle guidance software CBCT navigation technology accurately reaches ≤1 cm lesions Lower needle-repositioning times 29% smaller skin dose than conventional CT
	XperGuide ablation	Tumor ablations from adjacent tissues Customizable isotherm imaging for radiofrequency, microwave, and cryoablation Updates interactively during ablation
	EmboGuide	Automatic detection and treatment of tumors and vessel suppliers 3D images overlaid on X-ray images for navigation during embolization
	Vision 2	3D live guidance software Navigates catheters, coils, and other devices during IR procedures
	TrackVision 2	Needle advancement via planned trajectories on live fluoroscopy Accurate registration of 3D trajectories to C-arm and table movements

**Table 3:** Summary of commonly available (MONITORING) procedural software used in interventional therapies.

**Category**	**Software**	**Main Features**
MONITORING	AneurysmFlow	Blood flow pattern visualization Generates new data with the mean aneurysm flow amplitude ratio
	Volume Viewer	Rich in 3D-image-processing tools User interface with wide viewing space Summary schedule with measurements as images
	Volume Viewer Innova	Plans needle trajectories via multi-oblique mode Wizard panel for multivolume segmentation workflow

**Table 4:** Summary of commonly available (INTUITIVE DISPLAY) procedural software used in interventional therapies.

**Category**	**Software**	**Main Features**
INTUITIVE DISPLAY	SmartCT Roadmap	Live 3D segmented images Catheter navigation via vessel structure synthetic visualization
	VesselNavigator	3D dataset overlaid on live fluoroscopy images Navigates complex vessels and structures Minimizes contrast-enhanced procedures
	HeartNavigator	Insights into calcification distribution Volume-rendered 3D heart images for TAVR and TAVI Automatic heart sectoring into anatomical structures Ideal X-ray display angles
	EP Navigator	Catheter navigation software Generates 3D images using pre-interventional CT images or CTA databases Generates accurate 3D images for segmented heart structure
	EchoNavigator	Live echo and X-ray fusion software Live 3D TEE with integrated X-rays during structural heart procedures
	FlightPlan for EVAR	Simplifies EVAR sizing and planning on MRA or CTA Zero-click bone removal and CTA abdominal vessel tracking Endograft sealing-zone analyses
	FlightPlan for Liver	97% sensitivity for liver tumor-feeding vessels One-click extraction of hepatic vasculature data
	Advantage 4D	Illustrates anatomical objects in motion Reduces structural deformation Highlights dynamic range of motion for precise radiotherapy assessment
	Embo ASSIST with Virtual Injection	Simulates embolization with virtual injection using Edison One-click vasculature split from CBCT acquisition Supports embolization strategy by tracking injection path
	EVARVision	Integrates 2D X-ray and 3D images from multiple modalities
	Stereo 3D	Combination of Vision 2, EVARVision, and TrackVision 2 Reconstructs 3D objects from two spatially sectioned fluoroscopic acquisitions Localizes devices during 3D anatomy without CBCT acquisition

**Table 5:** Summary of commonly available (POSTPROCEDURAL CHECK) procedural software used in interventional therapies.

**Category**	**Software**	**Main Features**
POSTPROCEDURAL CHECK	SmartPerfusion	Intensified fluoroscopic X-ray software Generates color-coded DSA images Locates treatment endpoint via perfusion-imaging technology
	Innova 3D	Reconstructs 3D volumes from rotational fluoroscopy Displays axial, sagittal, coronal, and oblique cross-sections
	Innova CT HD	Reconstructs 3D volumes from rotational fluoroscopy Generates detailed images with fine-quality cross-sections

## Abbreviations

IR, interventional radiology; 3D, three-dimensional; GMDN, global medical device nomenclature; CBCT, cone-beam CT; HCC, hepatocellular carcinoma; TACE, transarterial chemoembolization; DSA, digital subtraction angiography; DICOM, digital imaging and communications in medicine; AW, advantage workstation; MR, magnetic resonance; MRA, magnetic resonance angiography; PACS, picture-archiving and communication system; PET, positron emission tomography; CTA, computed tomography angiography; IVUS, intravascular ultrasound; PCI, percutaneous coronary intervention; MIP, maximum intensity projection; TAVR, transcatheter aortic valve replacement; TAVI, transcatheter aortic valve implantation; AF, atrial fibrillation; 3D-ATG, 3D rotational angiography; TEE, transesophageal echocardiography; FEX, fused echocardiographic/X-ray fluoroscopic imaging; CHD, congenital heart disease.
